# Draft genome assemblies of three *Ligilactobacillus salivarius* strains isolated from commercial layer hen litter

**DOI:** 10.17912/micropub.biology.002411

**Published:** 2026-09-11

**Authors:** Benjamin Brehm-Stecher, Ryan Donaldson, Charlie Fisher, William Jensen, Alex Kottmeyer, Paul Lueth, Matthew Lutz, Elizabeth Makovec, Simon Alexander Radl, Ainsley Wood, Madison Stoltz, Nick Peters, Lucille C. Jonas

**Affiliations:** 1 Department of Plant Pathology, Entomology and Microbiology, Iowa State University, Ames, IA, USA; 2 Immunobiology Graduate Program, Iowa State University, Ames, IA, USA; 3 Interdepartmental Microbiology Graduate Program, Iowa State University, Ames, IA, USA; 4 Department of Animal Science, Iowa State University, Ames, IA, USA

## Abstract

Direct-fed probiotics in poultry can improve animal nutrition and health while also competing with human foodborne pathogens for colonization of the gastrointestinal tract. The objective of this study was to perform whole-genome sequencing on lactic acid bacteria isolated from commercial chicken litter to characterize genetic markers associated with probiotic potential. Three *Ligilactobacillus salivarius* strains were selected for whole-genome sequencing using Oxford Nanopore Technology. The genomes contained genes associated with sialic acid utilization, bacteriocin production, and antimicrobial resistance. While these markers may indicate suitability as probiotics in poultry, further experimentation is needed to verify the functionality of the identified genes.

**Table 1. Overview of the genome assemblies generated in this study:** The raw sequencing data and genome assemblies are available in the NCBI BioProject PRJNA1449388. Genomes were annotated using the Prokaryotic Genome Annotation Pipeline (PGAP), and CheckM completeness and contamination were calculated (Parks et al., 2015; Tatusova et al., 2016).

**Table:** 

Genome ID:	BBS2	SR3	WJ4
Taxonomy	Ligilactobacillus salivarius	Ligilactobacillus salivarius	Ligilactobacillus salivarius
Isolation source	Chicken litter	Chicken litter	Chicken litter
No. of bps sequenced	120,063,089	81,727,582	86,521,781
No. of reads	125,350	86,601	97,147
NCBI raw read accession number	SRX32803745	SRX32803744	SRX32803743
Average coverage	55x	41x	43x
No. of contigs	4	14	37
Assembly N50 (bps)	1,711,922	202,620	64,338
Assembly L50	1	2	9
Assembly size (bps), [GC content]	2,158,836 [32.99%]	1,962,979 [32.91%]	1,983,786 [32.86%]
No. genes annotated	2,097	1,927	1,976
CheckM completeness	94.42%	93.14%	91.73%
CheckM contamination	3.38%	2.87%	2.09%
GenBank accession number	GCA_057004665.1	GCA_056933575.1	GCA_056933615.1
Alanine racemase (BLASTp homolog)	MHS1608993.1 (WP_255906516.1)	MHR6080405.1 (WP_255906516.1)	MHR6109652.1 (WP_255906516.1)
Sialidase (BLASTp homolog)	MHS1610039.1 (WP_003708613.1)	MHR6081797.1 (WP_003708613.1)	MHR6109824.1 (WP_003708613.1)
Bacteriocin synthesis cluster	MHS1610007.1, MHS1610008.1, MHS1610009.1, MHS1610010.1, MHS1610011.1	MHR6081764.1, MHR6081765.1, MHR6081766.1, MHR6081767.1, MHR6081768.1	MHR6109790.1, MHR6109791.1, MHR6109792.1, MHR6109793.1, MHR6109794.1

## Description

Probiotics, such as lactic acid bacteria (LAB), are microorganisms that provide health benefits for human and animal hosts (Khurajog et al., 2023). In the poultry industry, direct-fed probiotics may alter the poultry gut microbiota, potentially improving nutrient absorption, boosting the host immune response, and ameliorating overall animal health (Naeem and Bourassa, 2025). Additionally, probiotics can produce lactic acid, hydrogen peroxide, and bacteriocins, which directly inhibit the growth of foodborne pathogens, including *Campylobacter jejuni* and *Salmonella enteritidis* (Sirisopapong et al., 2023). The objective of this investigation was to isolate and sequence the genomes of lactic acid bacteria from commercial layer hen litter to determine if they harbor traits relevant to their function as probiotics.

Initially, eleven strains of lactic acid bacteria were isolated from commercial chicken litter. Using Sanger sequencing of the 16S rRNA genes, these isolates were identified as *Ligilactobacillus* , *Limosilactobacillus, Lactobacillus,* and *Aerococcus* species. Three *Ligilactobacillus* isolates (BBS2, SR3, WJ4) were selected for long-read whole-genome sequencing. A summary of relevant statistics for the generated genome assemblies is presented in Table 1. The assembly of WJ4 was comprised of 1,983,786 bps across 37 contigs. BBS2 had the largest assembly size: 2,158,836 bps across 4 contigs. Finally, the assembly for SR3 contained 14 contigs and was 1,962,979 bps. CheckM predicted completeness and contamination scores of 91.73% and 2.09% for WJ4, 93.14% and 2.87% for SR3, and 94.42% and 3.38% for BBS2, respectively. Tetranucleotide correlation searches (TCS) were performed on the assemblies to identify related genomes in JspeciesDB, and average nucleotide identity (ANI) was calculated using MUMmer (Richter et al., 2016). Notably, the most similar TCS genome hit (z-score > 0.99) differed across all three isolates. Isolate BBS2 was most similar to *Ligilactobacillus salivarius* strain NIAS840 (97.95% identity, 83.97% alignment), isolate SR3 to *L. salivarius* strain UCC118 (97.47% identity, 83.99% alignment), and isolate WJ4 to *L. salivarius* strain GJ-24 (98.21% identity, 85.94% alignment). For context, ANI between two genomes of the same species is typically greater than 95% and greater than 99% between those of the same strain (Jain et al., 2018; Rodriguez-R et al., 2023). ANIm comparisons between our isolates revealed greater similarity among themselves than to the reference genomes. BBS2 and SR3 were highly similar, sharing 99.97% identity, with alignment coverages of 91.03% and 100%, respectively. In contrast, WJ4 was most similar to SR3, showing 98.32% identity across 86.10% alignment coverage.

The Resistance Gene Identifier (RGI) on the Comprehensive Antibiotic Resistance Database (CARD) identified a putative glycopeptide resistance gene, *vanT* , in all three of the isolates (Mukiri et al., 2025; Wlodarski et al., 2025). This gene was annotated as an alanine racemase by the Prokaryotic Genome Annotation Pipeline (PGAP) and was identical across BBS2, SR3, and WJ4. When BLASTed against the ClusteredNR database on NCBI, the translated protein sequence was most similar to WP_255906516.1 (99.46% amino acid identity, 100% alignment), which was also an *L. salivarius* alanine racemase (Tatusova et al., 2016). Alanine racemase catalyzes the conversion of L-alanine to D-alanine, which is an essential component of peptidoglycan biosynthesis in *L. salivarius* (Asojo et al., 2014). Previous literature suggests that VanT evolved from regular alanine racemases, acquiring additional selectivity for serine while retaining activity on alanine, thereby conferring vancomycin resistance by altering peptidoglycan precursors (Meziane-Cherif et al., 2015). These findings warrant further validation of the alanine racemase protein activity in our isolates to determine whether they carry vancomycin resistance.

Another interesting feature of the genomes examined in this study is that BBS2, SR3, and WJ4 all contained a copy of a sialidase family protein. Bacterial sialidases facilitate the utilization of host-derived glycans by liberating terminal sialic acids, with implications for nutrient availability and bacterial adhesion in the gastrointestinal tract (Juge et al., 2016). Bacteria harboring sialidases are thought to have a competitive advantage over those lacking this activity in the gastrointestinal tract (Buzun et al., 2024). The most similar sialidase homolog to that of WJ4 was WP_003708613.1 (99.46% amino acid identity, 100% alignment), another *L. salivarius* sialidase family protein. Similarly, WP_003708613.1 was also homologous to the sialidase found in SR3 and BBS2, but to a lesser extent (98.78% amino acid identity, 100% alignment). As all three genomes (BBS2, SR3, and WJ4) encoded a sialidase, this may represent a conserved adaptation that contributes to persistence and colonization of *L. salivarius* within the poultry gastrointestinal tract.

Additionally, the assemblies of BBS2, SR3, and WJ4 all contained identical putative bacteriocin synthesis clusters (Table 1). Other strains of *L. salivarius* produce salivaricin, a bacteriocin that can inhibit the growth of several foodborne pathogens, including *C. jejuni* and *Listeria monocytogenes* (Elnar et al., 2025; Messaoudi et al., 2012). *L. salivarius* strains are differentiated into subtypes based on the structure of the SalP operon, which produces salivaricin (Elnar et al., 2025). The genetic distinction between subtypes is based on the presence of two transport proteins, LanT and HlyD, in which strains lacking these proteins are unable to export a functional salivaricin (Elnar et al., 2025). A search for LanT homologs (WP_081538429.1) in BBS2, SR3, and WJ4 was conducted using BLASTp; however, no hits were identified. The same was observed with HlyD (WP_081538428.1). These findings indicate that the highlighted gene cluster is unlikely to export a functional bacteriocin, although further work is needed to validate this.

## Methods

Lactic acid bacteria were isolated from litter samples of layer hens at the Robert T. Hamilton Poultry Teaching and Research Facility at Iowa State University. Litter samples were emulsified in saline solution, serially diluted, and plated onto Lactobacilli MRS agar (BD Difco, USA). Samples were grown in air-tight boxes with Anaeropaks (Mitsubishi Gas Chemical America, USA) to simulate anaerobic conditions and were then incubated at 37°C. Bacterial colonies were picked based on morphology and subcultured onto MRS agar for purity. To initially identify the isolates, DNA was extracted from colonies, and the 16S rRNA gene was amplified by PCR using Illusta PuReTaq Ready-to-go PCR beads (Cytiva, USA) with the primers: 616F (5'-AGAGTTGATCMTGGCTCAG-3') and 1492R (5'-GTTACCTTGTTACGACTT-3'). The PCR products were purified using the GeneJET PCR Purification Kit (Thermo Scientific, USA) and submitted to the Iowa State DNA facility for Sanger sequencing. The resulting sequences were compared against the NCBI 16S ribosomal RNA database using BLASTn.

Three isolates identified as *Ligilactobacillus salivarius* were submitted for whole-genome sequencing. Bacterial Genome Sequencing was performed by Plasmidsaurus using Oxford Nanopore Technology with custom analysis and annotation. The genomes were assembled using Autocycler with Flye v2.9.6, Hifiasm v0.25.0, and Plassembler v1.8.0, using default parameters for high-quality long-read data (Bouras et al., 2023; Cheng et al., 2021; Kolmogorov et al., 2019; Wick et al., 2025). Assemblies were subsequently rotated using dnaapler v1.3.0 and polished with Medaka v1.8.0 (Bouras et al., 2024; Oxford Nanopore Technologies, 2023). The Prokaryotic Genome Annotation Pipeline (PGAP) was used to predict and annotate protein-coding sequences, with which CheckM completeness and contamination were also calculated (Parks et al., 2015; Tatusova et al., 2016). The Bacterial and Viral Bioinformatics Resource Center (BV-BRC, accessed 05/2026) was utilized to identify and explore relevant traits within the genomes (Shukla et al., 2026). Using JspeciesWS , tetranucleotide correlation searches (TCS) were performed against JspeciesDB, and average nucleotide identity (ANI) was calculated using MUMmer (Richter et al., 2016). Additionally, the Resistance Gene Identifier (RGI) for the Comprehensive Antibiotic Resistance Database (CARD) was used to identify putative antimicrobial resistance genes (Mukiri et al., 2025; Wlodarski et al., 2025). To confirm the identity and identify homologs of genes of interest, translated protein sequences underwent a BLAST search against the ClusteredNR database on NCBI (Sayers et al., 2025).
